# Malaria risk factor assessment using active and passive surveillance data from Aceh Besar, Indonesia, a low endemic, malaria elimination setting with *Plasmodium knowlesi, Plasmodium vivax,* and *Plasmodium falciparum*

**DOI:** 10.1186/s12936-016-1523-z

**Published:** 2016-09-13

**Authors:** Herdiana Herdiana, Chris Cotter, Farah N. Coutrier, Iska Zarlinda, Brittany W. Zelman, Yusrifar Kharisma Tirta, Bryan Greenhouse, Roly D. Gosling, Peter Baker, Maxine Whittaker, Michelle S. Hsiang

**Affiliations:** 1 School of Public Health, University of Queensland, Brisbane, QLD Australia; 2 United Nations Children’s Fund (UNICEF), Aceh Field Office, Banda Aceh, Indonesia; 3 Malaria Elimination Initiative, Global Health Group, University of California San Francisco (UCSF), San Francisco, CA USA; 4 Eijkman Institute for Molecular Biology, Jakarta, Indonesia; 5 Department of Medicine, UCSF, San Francisco, CA USA; 6 College of Public Health, Medical and Veterinary Sciences, University of James Cook, Townsville, QLD Australia; 7 Department of Pediatrics, University of Texas Southwestern Medical Center, Dallas, TX USA; 8 Department of Pediatrics, UCSF, San Francisco, CA USA

**Keywords:** Risk factor, Passive surveillance, Active surveillance, Reactive case detection, Aceh Besar, Indonesia, Low-endemic setting, Malaria elimination, *Plasmodium knowlesi*, *Plasmodium vivax*, Mixed species

## Abstract

**Background:**

As malaria transmission declines, it becomes more geographically focused and more likely due to asymptomatic and non-falciparum infections. To inform malaria elimination planning in the context of this changing epidemiology, local assessments on the risk factors for malaria infection are necessary, yet challenging due to the low number of malaria cases.

**Methods:**

A population-based, cross-sectional study was performed using passive and active surveillance data collected in Aceh Besar District, Indonesia from 2014 to 2015. Malaria infection was defined as symptomatic polymerase chain reaction (PCR)-confirmed infection in index cases reported from health facilities, and asymptomatic or symptomatic PCR-confirmed infection identified in reactive case detection (RACD). Potential risk factors for any infection, species-specific infection, or secondary-case detection in RACD were assessed through questionnaires and evaluated for associations.

**Results:**

Nineteen *Plasmodium knowlesi*, 12 *Plasmodium vivax* and six *Plasmodium falciparum* cases were identified passively, and 1495 community members screened in RACD, of which six secondary cases were detected (one *P. knowlesi*, three *P. vivax*, and two *P. falciparum*, with four being asymptomatic). Compared to non-infected subjects screened in RACD, cases identified through passive or active surveillance were more likely to be male (AOR 12.5, 95 % CI 3.0–52.1), adult (AOR 14.0, 95 % CI 2.2–89.6 for age 16–45 years compared to <15 years), have visited the forest in the previous month for any reason (AOR 5.6, 95 % CI 1.3–24.2), and have a workplace near or in the forest and requiring overnight stays (AOR 7.9, 95 % CI 1.6–39.7 compared to workplace not near or in the forest). Comparing subjects with infections of different species, differences were observed in sub-district of residence and other demographic and behavioural factors. Among subjects screened in RACD, cases compared to non-cases were more likely to be febrile and reside within 100 m of the index case.

**Conclusion:**

In this setting, risk of malaria infection in index and RACD identified cases was associated with forest exposure, particularly overnights in the forest for work. In low-transmission settings, utilization of data available through routine passive and active surveillance can support efforts to target individuals at high risk.

## Background

The World Health Organization (WHO) has called for the ambitious commitment to eliminate malaria in 35 countries by 2030 [1]. All heads of states in the Asia Pacific region have committed to national malaria elimination by this date as well [2]. In the low-endemic phase that precedes the achievement of malaria elimination, and generally follows a higher transmission period, malaria programmes are faced with the challenge of a changing epidemiology of malaria. In this phase, an increasing proportion of malaria is imported and the high-risk group is often no longer young children and pregnant women, but rather tied to demographic and occupational risk factors specific to a local setting. Transmission also becomes more geographically focal, and more likely propagated through asymptomatic and non-falciparum infections [3].

In high malaria transmission settings, children under 5 years of age [4] and pregnant women [5] are the groups most vulnerable. Other risk factors in these settings include: low socio-economic status [6], low education level [7], poor quality and/or traditionally constructed housing [8], and low coverage and/or utilization of insecticide-treated bed nets (ITNs) or long-lasting insecticide-treated nets (LLINs), and/or indoor residual spraying (IRS) [9]. In contrast, risk factor assessments from low-transmission settings have shown risk factors as being an adult male, travel, outdoor exposure related to occupation, sleeping outdoors, and social activities [3, 10, 11]. However, findings from most of these studies have been extrapolated from epidemiological trends seen among cases identified through passive surveillance, and without control groups. Cross-sectional surveys are challenging to perform in low-transmission settings due to the low prevalence of infection [12] and case–control studies may not be easily implementable in operational settings. Further, there are limited data from multi-species low-transmission settings.

In order to address the challenge of malaria transmission that is more geographically focal and characterized by asymptomatic infection, the WHO recommends intensified surveillance in high-risk areas through epidemiologic investigation of symptomatic cases reported by health facilities (case investigation) as well as reactive case detection (RACD), which is the screening of household members and neighbours of index cases reported in passive surveillance. RACD aims to find any individuals with ‘secondary’, often asymptomatic infection in order to provide treatment to interrupt further transmission [13]. RACD is resource intensive and used widely [14], but what epidemiological or operational factors are associated with secondary-case detection is unclear, and likely context specific [10, 15–17].

In recent years, widespread scale-up of interventions such as ITNs, IRS, diagnostics, and effective anti-malarial drugs has led to significant declines in malaria burden in Indonesia, from over 1.3 million cases in 2006 [18] to 252,027 laboratory-confirmed cases in 2014 [19]. Among 513 districts, 213 districts (42 %) are certified as malaria free and 125 districts (24 %) have applied for certification of malaria elimination [20]. To achieve elimination in the remaining districts, intensified and targeted context-specific strategies will be needed, and an understanding of the risk factors for malaria infection is critical to inform strategic planning and implementation.

To address this challenge, a population-based, risk-factor assessment study was conducted in Aceh Besar, a district of Aceh Province that is aiming for malaria elimination and has a unique multi-species epidemiology that includes *Plasmodium vivax, Plasmodium falciparum*, and recently discovered *Plasmodium knowlesi* [21]. This cross-sectional study utilizing passive and active surveillance data was conducted to identify potential risk factors for: (1) any symptomatic or asymptomatic infection; (2) species-specific infection; and, (3) infection identified through RACD.

## Methods

### Study design

A cross-sectional study was performed utilizing prospectively collected data obtained through population-based passive and active malaria surveillance.

### Study site

**Fig. 1:**
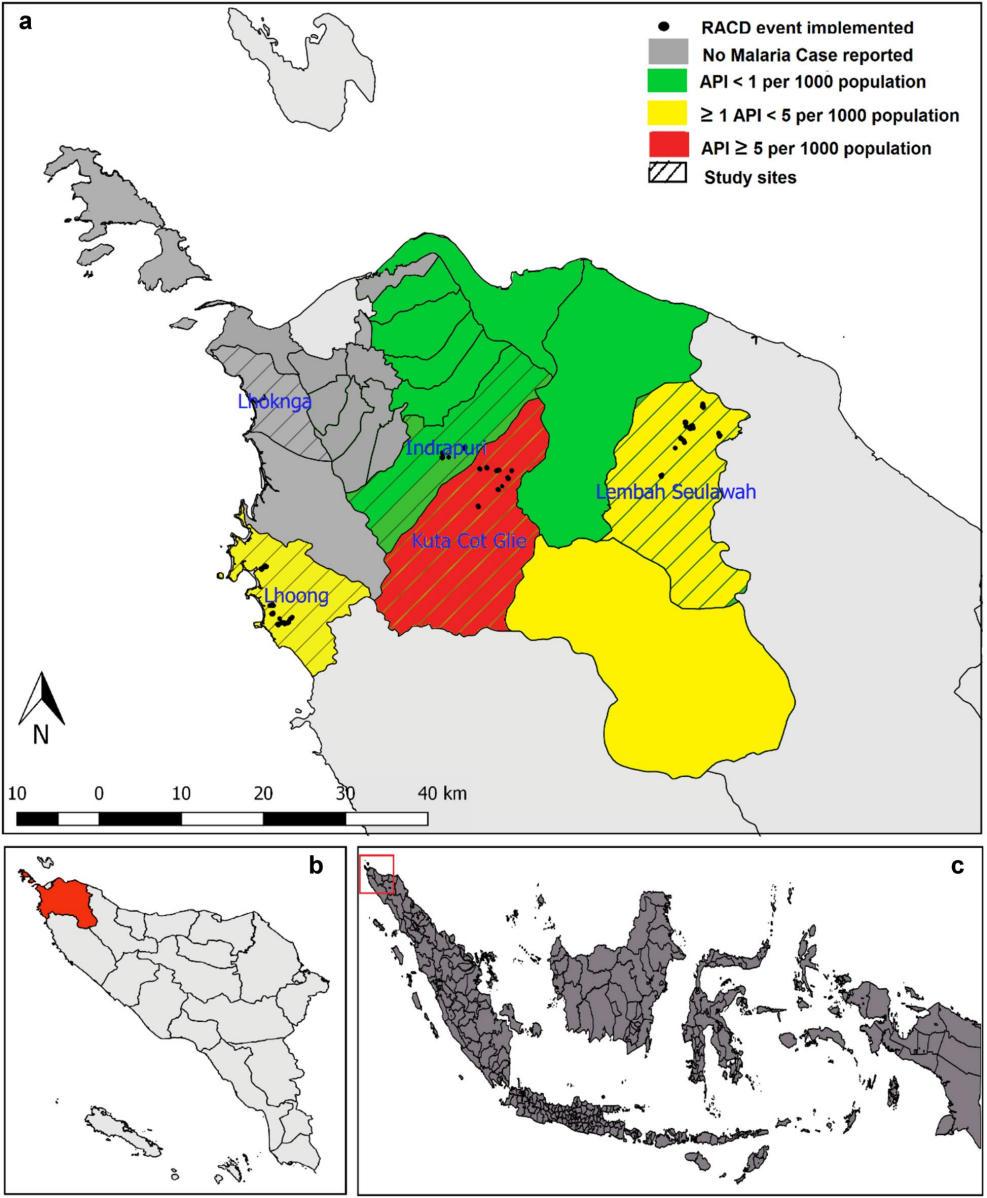
**a** Map of Aceh Besar District, with sub-district API rates from 2013 shown. Five study sub-districts shaded with *diagonal lines*. *Black dots* indicate locations of study RACD events. **b** Location of Aceh Besar in Aceh Province. **c** Location of Aceh Besar in Indonesia. *RACD* reactive case detection, *API* Annual parasite incidence

Aceh Besar is a district of Aceh Province, on Sumatra Island in western Indonesia (Fig. 1). The district covers 2903 km^2^ with most of its territory on Sumatra mainland plus some small islands. Being located near to the Equator, temperatures range from 25 to 29 °C and humidity is 65–85 %. From 2010 to 2014 monthly average rainfall ranged from 57.92 to 297 mm, with the rainy season spanning August to January [22, 23]. The high malaria transmission season occurs from January to July and August to December is considered the low transmission season. The annual parasite incidence (API) has been successfully reduced from 2.6 cases per 1000 population in 2006 [24], to 0.4 per 1000 in 2013 [25]. As part of an intensified malaria elimination strategy, in 2010, the District Health Office initiated case investigation and RACD, referred to as ‘contact survey’ in Indonesian. Designated malaria surveillance staff members based at health facilities visit the homes of all microscopy-confirmed malaria index cases reported from health facilities and perform an assessment to identify potential risk factors and classify the case as local or imported. Drawing on WHO guidelines and findings, the RACD policy involves microscopic diagnostic screening of all household members of an index case and of the neighbours living within a 500 m radius of the index case [26].

### Study population

Study population included malaria positive cases reported from five sub-district level primary health centres (PHCs) that reported 78 % of all cases reported in Aceh Besar in 2013: Indrapuri, Lembah Seulawah, Kuta Cot Glie, Lhoong, and Lhoknga, as well as household members and neighbours residing within a 500-m radius of index cases. The study was conducted over 19 months, from June 2014 to December 2015.

### Health facility and field procedures

Data collection for the study was nested within existing District Health Office passive and active surveillance procedures. Subjects presenting to PHCs with suspected malaria received a finger prick and blood was collected to prepare thick and thin smears for microscopy. Microscopy-confirmed cases were reported to district level within 1–3 days. At the time of diagnosis, informed consent was obtained for the collection of an additional 250 µL–3 mL of venous blood (depending on the age of the patient), which was partly used to generate dried blood spots (DBS), with the remaining stored as whole blood.

Within 1–3 days, the surveillance team visited the household of the index case to conduct a case investigation, which involved the administering of a structured questionnaire to assess potential risk factors. Within 1– 7 days of the index case report, RACD was conducted among subjects residing in households located within 500 m of the index cases. In RACD, blood was collected by finger prick and used to prepare thick and thin smears for microscopy as well as DBS, and household and individual level questionnaires similar to that used for the index case were administered. Questionnaires were translated into Bahasa Indonesia and administered using Nexus^®^ tablet computers. The household level questionnaire assessed socio-economic status, housing structure type, household population, proximity of house to a potential breeding site and to the forest, and bed net ownership, bed net usage the night prior, and IRS coverage in the past year. Questions assessing socio-economic status were based on the validated Indonesian basic health survey questionnaire [27]. The individual-level questionnaire assessed age, gender, travel history (defined as spending one night outside of their village in the previous month but not including the past week due to the incubation period of malaria being at least 1 week), occupation, frequency and reasons for visits to the forest in the last month, utilization of vector control measures such as bed nets, and history of having a fever within the previous 2 weeks. All positive cases detected at PHC or through RACD activities received malaria treatment based on slide and loop-mediated isothermal amplification (LAMP) results and according to national treatment guidelines [28].

The investigation team consisted of five people: a microscopist and surveillance officer from the PHC, two community health workers from the village (a midwife plus a village malaria worker or village leader), and the study field coordinator. Up to two return visits were conducted to maximize coverage of the target area, with a goal to reach at minimum 40 subjects or at least 80 % of the residents within each of the closest five households.

### Sample size

The study was part of umbrella study whereby the goal was to compare the yield of microscopy versus LAMP in RACD (anticipated positivity rates 2.5 and 5.0 %, respectively). Thus, the study was powered to compare paired proportions of LAMP versus microscopy positivity when the number screened in RACD reached 940 (α = 0.05, two-sided, β = 0.80), but with the plan to continue over at least a 1-year period to enable analyses to take into account seasonal variation.

### Laboratory testing

In passive surveillance, thick and thin blood smears were collected from suspected malaria cases and examined immediately by trained microscopists at the PHCs. In RACD, PHC microscopists collected thick and thin blood smears in the field and transported them in closed slide boxes for subsequent examination at the PHC. Slides were fixed and stained with 3 % Giemsa. Parasite densities were determined by counting the number of asexual parasites per 200 or 500 white blood cells (WBC) and calculating parasites/µL assuming a WBC count of 8000 parasites/µL. A thin smear was considered negative if no parasites were seen in 100 high-powered fields. If positive, an additional 100 high-powered fields were examined to determine species [29]. Quality assurance (QA) was conducted by a district expert level-certified microscopist for all positive cases and 10 % of randomly selected negatives. Further cross-checking was carried out at provincial level for all positive cases and to resolve discrepant results between the PHC and district-level microscopists [13].

All DBS samples collected were dried overnight then stored in sealed plastic bags with desiccant. Initial extraction of DNA and LAMP testing on all samples were performed at the Aceh Provincial Health Laboratory. DNA was extracted from DBS using the Saponin/Chelex-100 method [30]. Using 15 µL of chelex-extracted DNA, Pan-LAMP testing followed by Pf-LAMP specific testing for Pan-LAMP positive samples was performed using a commerical Loopamp detection kit [31, 32] in accordance to manufacturer’s instructions (Eiken Chemical, Japan). Pan-LAMP positive cases underwent further molecular testing at the Eijkman Institute using DNA that was chelex-extracted from a second DBS. Samples first underwent nested PCR targeting the *cytochrome b* gene, followed by *Alu*I enzyme digestion for species identification (not including *P. knowlesi*) as previously described [33–35]. After a report of indeterminate species and suspicion of *P. knowlesi* by a field microscopist, as well as limited data on the performance of the *cytochrome b* PCR method for detection of *P. knowlesi*, additional methods were employed. All Pan-LAMP positive samples were then tested by 18S rDNA nested PCR [36, 37] as well as *P. knowlesi*-specific PCR [38]. For a proportion of those testing positive by *P. knowlesi*-specific PCR, Qiagen-extracted DNA from whole blood (QIAamp Blood Minikit, Qiagen, CA) underwent sequencing (Eijkman Institute Sequencing Facility). All extractions were performed in rooms separate from where amplification was conducted. Using chelex-extracted DNA from a second blood spot, PCR targeting the *cytochrome b* gene was also performed on 10 % of randomly selected LAMP negative individuals from RACD.

DBS were stored at 4 °C within 1 week of collection, and then at −20 °C within 1 month of collection. Venous blood samples were stored at 4 °C for up to 5 days after collection, then transported along with the second blood spot in an ice box to Eijkman Institute in Jakarta using a 1-day service. Upon arrival at Eijkman, samples underwent DNA extraction or were stored at −20 °C (DBS) or −80 °C (whole blood samples). All extracted DNA was stored at −20 °C.

### Data management and statistical analysis

Data were merged, cleaned and analysed using STATA version 14.0. Maps were generated using QGIS version 2.14.0.

The primary analysis was to evaluate the associations between potential risk factors and any PCR-confirmed symptomatic or asymptomatic infection. Secondary analyses were to evaluate the associations between potential risk factors and PCR-confirmed species-specific infection, as well as PCR-confirmed infection identified through RACD.

To explore relationships between potential risk factors and each of the outcome measures, the Chi squared test or Fisher’s exact test was used for categorical variables, and Wilcoxon rank-sum or Kruskal–Wallis rank test was used for continuous variables. P values less than 0.05 were considered statistically significant, and further explored in a bivariate mixed effects logistic regression model. For the multivariate mixed effects logistic regression model, variables with P values <0.25 in the unadjusted analysis were considered [39] and correlation and collinearity across all variables was considered. A purposeful selection approach was used to select the model of best fit. Variables that had P value <0.1 and changed any parameter estimates by 20 % were retained [40]. Akaike’s information criteria (AIC) and likelihood ratio test (LRT) were also taken into consideration in the model selection [41]. The bivariate and multivariate models accounted for clustering at the level of the household, and because of the small sample size for secondary outcome measures, they were only performed for the primary analysis.

Independent variables were divided into three categories: socio-demographic, prevention and behaviours, and forest exposure and environment (Tables 1, 2, 3). Occupation was originally recorded as one of 14 types. After preliminary analyses identified forest-related work as a major risk factor for infection, one constructing variable was developed by re-classifying occupation into three sub-groups: unemployed; non forest-related jobs including: student, fisherman, manufacturing/factory, office/clerical, housewife, retail/shopkeeper, and driver; and, forest-related jobs that included logger, soldier/police, farmer, rubber tapper, miner, and other forest worker). The quality of housing structure variable was constructed based on type of window, wall, floor, and roof. Good quality was defined as having a window, cement wall, cement or tile floor, and tin roof; poor quality was defined as no window, wood wall, dirt floor, and no roof or having a thatched roof. Houses not meeting these criteria were otherwise considered moderate quality. The wealth index was generated by multiple correspondence analysis (MCA) based on 11 assets ownership variables for binary and categorical data [42]. The MCA output was taken as a weight for each variables and wealth index was constructed based on the first dimension that explained 70 % by MCA output. Wealth index was then ranked into quintiles, with the wealthiest represented in the highest quintile. For the secondary case analysis, index case and RACD-level factors were also assessed including: distance to the index case household, *Plasmodium* species, RACD response time from date of index case presentation, and coverage of RACD among all eligible individuals in the intervention area.

**Table 1:** Distribution (%) of potential risk factors among malaria cases (index and secondary cases), non-cases (screened in RACD), and by *Plasmodium* types

Variable	Non-cases no. (%) (n = 1489)	Cases no. (%) (n = 43)	*P*	Cases by *Plasmodium* type			
				Pf no. (%) (n = 8)	Pv no. (%) (n = 15)	Pk no. (%) (n = 20)	*P*
Detection method							
Passive	n/a	37 (100)	<0.0001	6 (16.2)	12 (32.4)	19 (51.4)	0.253
RACD	1489 (99.6)	6 (0.4)		2 (33.3)	3 (50.0)	1 (16.7)	
Socio-demographic							
Age category (years)							
≤15	481 (99.6)	2 (0.4)	<0.0001	0 (0.0)	0 (0.0)	2 (100)	0.033
16–30	362 (94.3)	22 (5.7)		4 (18.2)	12 (54.6)	6 (27.3)	
31–45	356 (94.9)	19 (5.1)		4 (21.0)	3 (15.8)	12 (63.2)	
46 +	290 (100)	0 (0.0)		0 (0.0)	0 (0.0)	0 (0.0)	
Gender							
Female	836 (99.6)	3 (0.4)	<0.0001	0 (0.0)	0 (0.0)	3 (100)	0.281
Male	653 (94.2)	40 (5.8)		8 (20.0)	15 (37.5)	17 (42.5)	
Education of household head							
No school	40 (95.2)	2 (4.8)	0.072	0 (0.0)	0 (0.0)	2 (100)	0.006
Primary school	481 (98.0)	10 (2.0)		1 (10.0)	8 (80.0)	1 (10.0)	
Secondary school	670 (96.1)	27 (3.9)		7 (25.9)	7 (25.9)	13 (48.2)	
Tertiary school	298 (98.7)	4 (1.3)		0 (0.0)	0 (0.0)	4 (100)	
Occupation category							
Unemployed	281 (99.7)	1 (0.3)	<0.0001	0 (0.0)	1 (100)	0 (0.0)	0.112
Not forest-related job	847 (98.6)	12 (1.4)		1 (8.3)	2 (16.7)	9 (75.0)	
Forest-related job	361 (92.3)	30 (7.7)		7 (23.3)	12 (40.0)	11 (36.7)	
Wealth index (quintile)							
1st	295 (96.1)	12 (3.9)	0.517	3 (25.0)	2 (16.7)	7 (58.3)	0.470
2nd	306 (97.8)	7 (2.2)		1 (14.3)	3 (42.9)	3 (42.9)	
3rd	302 (97.4)	8 (2.6)		3 (37.5)	3 (37.5)	2 (25.0)	
4th	444 (97.8)	10 (2.2)		1 (10.0)	3 (30.0)	6 (60.0)	
5th	142 (95.9)	6 (4.1)		0 (0.0)	4 (66.7)	2 (33.3)	
Subdistrict							
Lembah Seulawah	525 (97.2)	15 (2.8)	0.978	2 (13.3)	1 (6.7)	12 (80.0)	<0.0001
Kuta Cot Glie	374 (97.4)	10 (2.6)		6 (60.0)	1 (10.0)	3 (30.0)	
Lhoong	511 (97.0)	16 (3.0)		0 (0.0)	12 (75.0)	4 (25.0)	
Indrapuri	79 (97.5)	2 (2.5)		0 (0.0)	1 (50.0)	1 (50.0)	
Lhoknga	0 (0.0)	0 (0.0)		0 (0.0)	0 (0.0)	0 (0.0)	
Prevention and behaviours							
Travel							
No	1340 (98.6)	19 (1.4)	<0.0001	1 (5.3)	6 (31.6)	12 (63.2)	0.071
Yes	149 (86.1)	24 (13.9)		7 (29.2)	9 (37.5)	8 (33.3)	
ITN ownership							
No ITN	499 (96.2)	20 (3.8)	0.119	4 (20.0)	6 (30.0)	10 (50.0)	0.911
<1 ITN/2 people	712 (98.1)	14 (1.9)		2 (14.3)	5 (35.7)	7 (50.0)	
≥1 ITN/2 people	278 (96.9)	9 (3.1)		2 (22.2)	4 (44.4)	3 (33.3)	
Slept under a bed net previous night							
No	658 (96.0)	27 (4.0)	0.016	7 (25.9)	9 (33.3)	11 (40.7)	0.264
Yes	831 (98.1)	16 (1.9)		1 (6.3)	6 (37.5)	9 (56.3)	
House sprayed in previous 1 year							
No	1381 (97.2)	40 (2.8)	0.945	8 (20.0)	13 (32.5)	19 (47.5)	0.575
Yes	108 (97.3)	3 (2.7)		0 (0.0)	2 (66.7)	1 (33.3)	
Slept outside house previous night							
No	1459 (97.1)	43 (2.9)	0.347	8 (18.6)	15 (34.9)	20 (46.5)	n/a
Yes	30 (100)	0 (0.0)		0 (0.0)	0 (0.0)	0 (0.0)	
Forest exposure and environment							
Workplace near or in forest							
No	1027 (99.2)	8 (0.8)	<0.0001	0 (0.0)	3 (37.5)	5 (62.5)	0.401
Yes, overnights not required	273 (98.6)	4 (1.4)		0 (0.0)	1 (25.0)	3 (75.0)	
Yes, overnights required	189 (85.9)	31 (14.1)		8 (25.8)	11 (35.5)	12 (38.7)	
Distance workplace to forest (km)^a^							
In forest	209 (87.8)	29 (12.2)	<0.0001	7 (24.1)	12 (41.4)	10 (34.5)	0.373
<1	191 (98.0)	4 (2.0)		1 (25.0)	0 (0.0)	3 (75.0)	
1–5	59 (98.3)	1 (1.7)		0 (0.0)	0 (0.0)	1 (100)	
>5–10	3 (75.0)	1 (25.0)		0 (0.0)	0 (0.0)	1 (100)	
Visited forest in the last month for any reason							
No	1241 (99.0)	12 (1.0)	<0.0001	1 (8.3)	4 (33.3)	7 (58.3)	0.552
Yes	248 (88.9)	31 (11.1)		7 (22.6)	11 (35.5)	13 (41.9)	
Main reason^b^							
Residence	108 (94.7)	6 (5.3)	0.029	0 (0.0)	1 (16.7)	5 (83.3)	0.032
Work	111 (84.1)	21 (15.9)		6 (28.6)	10 (47.6)	5 (23.8)	
Other reason^c^	29 (87.9)	4 (12.1)		1 (25.0)	0 (0.0)	3 (75.0)	
Median visit (days), range^b^	30 (1–30)	14 (1–30)	0.002	14 (4–30)	10 (1–30)	25 (3–30)	0.603
Malaria transmission season							
Low	717 (97.6)	18 (2.4)	0.415	3 (16.7)	6 (33.3)	9 (50.0)	0.921
High	772 (96.9)	25 (3.1)		5 (20.0)	9 (36.0)	11 (44.0)	
Reported living near water body							
No	633 (98.0)	13 (2.0)	0.108	3 (23.1)	2 (15.4)	8 (61.5)	0.204
Yes	856 (96.6)	30 (3.4)		5 (16.7)	13 (43.3)	12 (40.0)	
Distance living to water bodyd (m)							
<100	435 (96.0)	18 (4.0)	0.609	2 (11.1)	8 (44.4)	8 (44.4)	0.563
100–499	168 (97.1)	5 (2.9)		2 (40.0)	1 (20.0)	2 (40.0)	
≥500	253 (97.3)	7 (2.7)		1 (14.3)	4 (57.1)	2 (28.6)	
Reported living near or in forest							
No	712 (97.4)	19 (2.6)	0.638	4 (21.1)	8 (42.1)	7 (36.8)	0.566
Yes	777 (97.0)	24 (3.0)		4 (16.7)	7 (29.2)	13 (54.2)	
Distance living to foreste (km)							
In forest	147 (96.1)	6 (3.9)	0.651	0 (0.0)	1 (16.7)	5 (83.3)	0.040
<1	440 (96.9)	14 (3.1)		3 (21.4)	3 (21.4)	8 (57.1)	
1-5	170 (98.3)	3 (1.7)		0 (0.0)	3 (100)	0 (0.0)	
>5 and <10	20 (95.2)	1 (4.8)		1 (100)	0 (0.0)	0 (0.0)	
Housing quality, composite variable							
Good	831 (97.7)	20 (2.4)	0.162	3 (15.0)	7 (35.0)	10 (50.0)	0.181
Moderate	335 (95.7)	15 (4.3)		1 (6.7)	7 (46.7)	7 (46.7)	
Poor	323 (97.6)	8 (2.4)		4 (50.0)	1 (12.5)	3 (37.5)	

^a^n = 497 people (462 non-cases and 35 cases: eight *P. falciparum*, 12 *P. vivax*, 15 *P. knowlesi*)

^b^n = 279 people (248 non-cases and 31 cases: seven *P. falciparum*, 11 *P. vivax*, 13 *P. knowlesi*)

^c^Other reasons: farming, hunting, school, accompanying parent

^d^n = 886 people (856 non-cases and 30 cases: five *P. falciparum*, 13 *P. vivax*, 12 *P. knowlesi*)

^e^n = 801 people (777 non-cases and 24 cases: four *P. falciparum*, seven *P. vivax*, 13 *P. knowlesi*)

**Table 2:** Bivariate and multivariate analyses among malaria infected (n = 43) versus non-infected subjects (n = 1489)

Variables	Bivariate		Multivariable	
	OR (95 % CI)	*P*	AOR (95 % CI)	*P*
Socio-demographic				
Age category (years)	≤15 as reference			
16–45	15.17 (3.55–64.8)	<0.0001	13.98 (2.17–89.58)	0.005
46+^a^	–	–	–	–
Gender	Female as reference			
Male	20.26 (5.97–68.69)	<0.0001	12.54 (3.02–52.12)	0.001
Occupation category	Unemployed as reference			
Not forest-related job	4.06 (0.52–31.99)	0.183	–	–
Forest-related job	28.87 (3.76–221.64)	0.001	–	–
Prevention and behaviours				
Travel in the previous month	No as reference			
Yes	42.78 (14.29–128.20)	<0.0001	–	–
Sleep under bed net	No as reference			
Yes	0.45 (0.23–0.90)	0.023	2.75 (0.83–9.05)	0.097
Forest exposure and environment				
Workplace near or in forest	No as reference			
Yes, overnights not required	2.20 (0.55–8.72)	0.263	0.60 (0.11–3.21)	0.555
Yes, overnights required	96.63 (23.00–406.21)	<0.0001	7.92 (1.58–39.71)	0.012
Visited forest in previous month for any reason	No as reference			
Yes	35.23 (11.54–107.58)	<0.0001	5.62 (1.31–24.15)	0.020

^a^Category dropped as no malaria infected subjects in this age category

**Table 3:** Distribution and associations between epidemiological and operational factors among secondary case and non-secondary case from RACD

Variable	Non-secondary case (n = 1489)		Secondary case (n = 6)		*P*
	No.	(%)	No.	(%)	
Socio-demographic					
Age category (years)					
≤15	481	99.8	1	0.2	0.151
16–30	362	98.9	4	1.1	
31–45	356	99.7	1	0.3	
46+	290	100	0	0.0	
Gender					
Female	836	100	0	0.0	0.007
Male	653	99.1	6	0.9	
Education of household head					
No school	40	100	0	0.0	0.450
Primary school	481	99.8	1	0.2	
Secondary school	670	99.3	5	0.7	
Tertiary school	298	100	0	0.0	
Occupation category					
Unemployed	281	100	0	0.0	0.010
Not forest-related job	847	99.9	1	0.1	
Forest-related job	361	98.6	5	1.4	
Wealth index ( quintile)					
1st	295	99.7	1	0.3	0.796
2nd	306	99.7	1	0.3	
3rd	302	99.3	2	0.7	
4th	444	99.8	1	0.2	
5th	142	99.3	1	0.7	
Sub-district					
Lembah Seulawah	525	99.4	3	0.6	0.917
Kuta Cot Glie	374	99.7	1	0.3	
Lhoong	511	99.6	2	0.4	
Indrapuri	79	100	0	0.0	
Lhoknga	n/a	n/a	n/a	n/a	
Clinical history					
Reported fever in previous 2 weeks					
No	1445	99.7	4	0.3	0.013
Yes	44	95.7	2	4.3	
Prevention and behaviours					
Travel					
No	1340	99.8	3	0.2	0.016
Yes	149	98.0	3	2.0	
Bed net ownership					
No ITN	499	99.2	4	0.8	0.289
<1 ITN/2 people	712	99.7	2	0.3	
≥1 ITN/2 people	278	100	0	0.0	
Slept under bed net previous night					
No	658	99.3	5	0.7	0.094
Yes	831	99.9	1	0.1	
House spraying in previous 1 year					
No	1381	99.6	6	0.4	0.493
Yes	108	100	0	0.0	
Slept outside house last night					
No	1459	99.6	6	0.4	0.725
Yes	30	100	0	0.0	
Forest exposure and environment					
Workplace near or in forest					
No	1027	100	0	0.0	<0.0001
Yes, overnights not required	273	100	0	0.0	
Yes, overnights required	189	96.9	6	3.1	
Distance workplace to forest (n = 462)					
In forest	209	97.2	6	2.8	0.085
<1 km	191	100	0	0.0	
1–5 km	59	100	0	0.0	
>5–10 km	3	100	0	0.0	
Visited forest in the last month					
No	1241	99.9	1	0.1	0.001
Yes	248	98.0	5	2.0	
Main reason (n = 253)					
Residence	108	99.1	1	0.9	0.430
Work	111	96.5	4	3.5	
Other reason	29	100	0	0.0	
Median visit (days), range (n = 253)	30 (1–30)	–	14 (4–30)	–	0.596
Season					
Low transmission	734	99.7	2	0.3	0.688
High transmission	793	99.5	4	0.5	
Reported living near water bodies					
No	633	99.7	2	0.3	0.650
Yes	856	99.5	4	0.5	
Reported living near or in forest					
No	712	99.7	2	0.3	0.689
Yes	777	99.5	4	0.5	
Housing quality					
Good	831	99.6	3	0.4	0.858
Moderate	335	99.4	2	0.6	
Poor	323	99.7	1	0.3	
RACD and index case level factors					
Distance from home of index case					
At the same house	203	98.1	4	1.9	0.020
<100 m	926	99.8	2	0.2	
100–499 m	319	100	0	0.0	
≥500 m	41	100	0	0.0	
Population coverage of RACD					
<90 %	429	99.1	4	0.9	0.062
≥90 %	1060	99.8	2	0.2	
Time from report of index case to RACD					
At the same day	45	100	0	0.0	0.804
1–7 days	1388	99.6	6	0.4	
>7 days	56	100	0	0.0	
Age category of index case (years)					
≤15	18	100	0	0.0	0.715
16–30	770	99.5	4	0.5	
30–45	655	99.7	2	0.3	
Gender of index case					
Female	63	100	0	0.0	0.607
Male	1426	99.6	6	0.4	
Occupation category of index case					
Not forest-related job	393	99.5	2	0.5	0.658
Forest-related job	1096	99.6	4	0.4	

## Results

### Enrolment

**Fig. 2:**
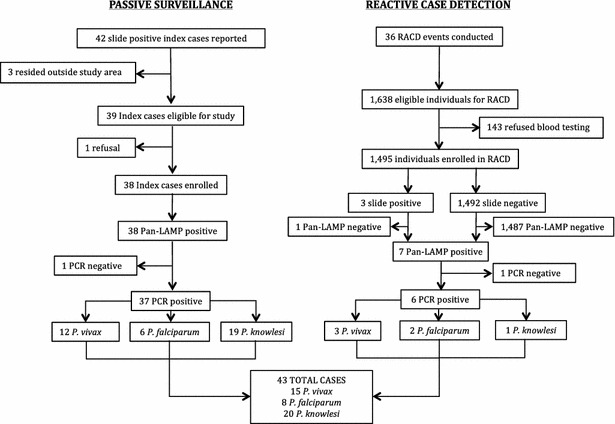
Study recruitment and laboratory testing results 38 index cases were enrolled through passive surveillance and triggered 36 RACD events. One RACD event covered three contemporaneous indexes cases from the same household. In passive surveillance, 37 cases were confirmed by PCR and by RACD, there were six PCR-confirmed cases, resulting in a total of 43 cases. *Pan-LAMP* Pan-loop-mediated isothermal amplification

Of 42 index cases reported in passive surveillance, three were excluded due to residence outside the study area. Of the 39 index cases eligible for case investigation, one was a relapse that occurred 10 weeks after the initial infection and the index case refused case investigation (Fig. 2). The remaining 38 index cases triggered 36 RACD events in 33 villages. One RACD event was conducted to cover three indexes cases that resided in the same household and were reported within 3 days of each other. In total, there were 1638 household members and neighbours (excluding index cases) who resided in the targeted screening area, of whom 1495 (91.3 %) were interviewed and provided blood for malaria testing after providing informed consent. The refusal rate for blood testing was 8.7 % (143/1638) with 81 % of refusals coming from adult men (116/143). Reasons for refusal included: afraid of pain (83), not interested (50), no reason provided (26), and child too young (19). On average, one return visit was required to reach 100 % of villagers. No eligible subjects were missed, likely because targeted households were sensitized by the village midwife, a village leader or a malaria volunteer prior the date of RACD event.

### Laboratory testing results

Of 42 slide-positive malaria cases reported through passive surveillance, 41 were ultimately confirmed microscopy positive on the second read (16 *P. vivax*, 20 *P. falciparum*, two *P. knowlesi*, and three *Plasmodium malariae*). Among 38 microscopy-confirmed index cases enrolled in the study, 37 were Pan-LAMP and PCR confirmed and species identification using molecular methods identified 12 *P. vivax*, six *P. falciparum* and 19 *P. knowlesi* infections. In RACD, three of 1495 tested positive by microscopy (one *P. vivax* and two *P. falciparum*). After subsequent testing by Pan-LAMP and PCR, a total of six PCR-confirmed malaria cases were identified as three *P. vivax*, two *P. falciparum* and one *P. knowlesi*. Pan-LAMP positive but PCR negative cases included one index case that was identified as *P. vivax* by microscopy but DBS was collected after treatment, and one microscopy negative case from RACD. Combining passive and active surveillance data, a total of 43 malaria cases were identified, including 15 (34.9 %) *P. vivax*, eight (18.6 %) *P. falciparum* and 20 (46.5 %) *P. knowlesi* (Fig. 2). Of note, *P. knowlesi* infections were misdiagnosed by microscopy as ten *P. falciparum*, seven *P. vivax*, one *P. malariae*, and two indeterminate). Non-cases for the risk factor analyses included 1489 RACD subjects who were Pan-LAMP or PCR negative. Ten percent of Pan-LAMP negative RACD samples were randomly selected for quality assurance and all were confirmed negative by PCR. Additional details of the laboratory testing results have been reported elsewhere [21].

### Characteristics of the overall study population

Most study participants were female (54.7 %) (Table 1). The highest proportion of subjects (31.5 %) fell in the youngest age group (≤15 years), followed by 16–30 years (25.1 %), 31–45 years (24.5 %), and ≥46 years (18.9 %). Most of household heads had secondary school-level education (45.5 %). A quarter of study participants had forest-related jobs: farming (16.4 %), logging (3.2 %), soldier/police (0.5 %), mining (0.3 %), and other forest jobs (5.2 %). Of those reporting a workplace near or in the forest, 71.4 % were required to stay overnight. Overall, bed net usage the night prior was 55.2 % and IRS coverage in the past year was 7.3 %. More than half of study participants (57.8 %) reported living near a water body, and in most, the water body was located within 100 m. More than half of participants (52.3 %) reported living near or in the forest. Most houses (55.6 %) were good quality. More than 95 % of malaria cases occurred in three of the five sub-districts and malaria cases were reported in both the historically high (57.2 %) and low transmission seasons (42.8 %).

### Characteristics of RACD events and index cases triggering RACD

Index cases were generally adult males with forest-related jobs. Of RACD events, 96.3 % were conducted within 7 days of the index case report. Among 71 % of RACD events, coverage (participation rate) was at least 90 %. The mean distance of RACD screening events conducted from the index case household was 91 m (range: 0–1897 m). Most individuals screened in RACD (75.9 %) resided within 100 m of the index cases. Forty-one subjects (2.7 %) screened resided more than 500 m from the index case but were included due to there being fewer than five households within 500 m of the index case.

### Assessment of risk factors for malaria infection

Table 1 shows the distribution of potential risk factors among malaria-infected versus uninfected subjects analysed using Chi squared or Fisher’s exact test and methods for non-parametric variables to explore the associations. There were significant associations between malaria infection and age category, gender, occupation category, travel, sleeping under a bed net last night, workplace location near or in the forest (including required overnights and distance of workplace to forest for those with a workplace near or in the forest), and reported visits to the forest in the previous month for any reason.

The results of bivariate and best-fit multivariable analysis with household level adjustment for all symptomatic and asymptomatic malaria infection identified in passive or RACD are presented in Table 2. In the final multivariable model, adult age, male gender, workplace near or in the forest requiring overnight stays, and visits to the forest in previous month for any reason were significantly associated with malaria infection. Adults aged 16–45 years had 14.0 times higher odds (95 % CI 2.2–89.6, P = 0.005) of malaria infection compared to the reference group of age ≤15 years. Males had 12.5 times higher odds infection compared to females (95 % CI 3.0–52.1, P = 0.001). Individuals with a workplace location in or near the forest and requiring overnight stays had a 7.9 times higher odds of infection compared to those whose workplace was not in or near the forest (95 % CI 1.6–39.7, P = 0.012). Individuals who visited forest in previous month for any reason had a 5.6 times higher odds of infection compared to those who had not visited the forest (95 % CI 1.3–24.2, P = 0.020).

### Assessment of risk factors for *Plasmodium falciparum* versus *Plasmodium vivax* versus *Plasmodium knowlesi* infection

There were eight *P. falciparum*, 15 *P. vivax* and 20 *P. knowlesi* infections. The finding of *P. knowlesi* was unexpected as only *P. falciparum* and *P. vivax* had been reported from Aceh Besar in the past, and in Indonesia, *P. knowlesi* had only previously been reported from Borneo [43, 44]. As the epidemiology of species-specific infections can vary, a secondary aim of the study was to identify risk factors for infection by specific *Plasmodium* types. After the finding of *P. knowlesi* representing almost half of the infections, the local programme was especially interested to understand specific risk factors for *P. knowlesi* compared to other species and with negative. The sample sizes were too small to justify logistic regression analyses, but characteristics of subjects with different species of infection were compared and the relationships between potential risk factors and *P. knowlesi*, *P. vivax*, or *P. falciparum* infection (compared to non-cases) were explored using Chi squared or Fisher’s exact test or non-parametric methods.

In the comparison of *P. knowlesi* cases to non-cases, *P. vivax* cases to non-cases, and *P. falciparum* cases to non-cases, the findings were overall similar to the primary analysis in that adult age, male gender, forest-related work, travel, overnights in the forest for work, and forest exposure for any reason were associated with each of the infections (data not shown). In the comparison of the potential risk factors between the different species of infection, *P. knowlesi* cases were more likely to also have forest exposure from residence and other reasons, versus work only (Table 1). Compared to *P. knowlesi* subjects, those with *P. vivax* infection or *P. falciparum* were more likely to report travel (at least one night outside of their village in the previous month but not including the previous week). Infections were more likely in male adults for all species, though the only three malaria cases in females and/or children were due to *P. knowlesi* and all had forest exposures. The three female *P. knowlesi* cases included two students (aged 8 and 12 years) and a 32 years old teacher. Two of the female *P. knowlesi* cases had forest exposures: one reported that her workplace was within 1 km of forest and another had visited her parent who worked at the forest. One child did not live or work in or near the forest, but lived with another *P. knowlesi* case and there was a captive monkey at the home, thought to be the source of the infection.

*Plasmodium falciparum* cases and *P. knowlesi* cases were more likely to have a household head with secondary-level education, while *P. vivax* cases were more like to come from households with lower education status. In terms of geography, *Plasmodium* types clustered by sub-district. *Plasmodium falciparum* cases were most likely in Kuta Cot Glie. *Plasmodium vivax* cases were most likely detected in Lhoong, and *P. knowlesi* cases were mostly in Lembah Seulawah. All *Plasmodium* types were identified by passive surveillance and RACD, however in passive surveillance most infections were due to *P. knowlesi* (51.4 %), and in RACD, half of secondary cases were asymptomatic *P. vivax* cases (3/6), with the other asymptomatic case being a *P. falciparum* case.

### Assessment of factors associated with secondary case detection

In the risk factor analysis among subjects screened in RACD (Table 3), the two most notable findings were that compared to non-cases, secondary cases were more likely to have fever (4.3 vs. 0.3 % without fever, P = 0.013) and reside in the same household or within 100 m of the index case (100 vs. 0 % residing ≥100 m, P = 0.02). Among subjects residing in the same households as the index case, 1.9 % (4/207) were infected. The positivity rate dropped beyond the index case house, with 0.2 % (2/928) being infected within 100 m, and none beyond 100 m.

The relationship between secondary infection and a variety of other operational and index case-level factors were assessed. These included population coverage of the RACD event, response time, age, gender, or occupation of the index case, and there were no other significant relationships. There was also no association between high versus low malaria transmission season and finding secondary cases. As for other potential individual level risk factors, findings were similar to those in the overall primary analysis. Compared to 1489 non-cases, the six secondary cases were more likely to be male, report recent travel, be employed in forest-related work, have a workplace in or near the forest, and have visited the forest for any reason in the prior month.

## Discussion

The primary risk factors for malaria infection in the low endemic, multi-species area of Aceh Besar District were adult age, male gender and forest exposures, particularly related to forest-related occupation requiring overnight stays. There were some differences in demographic and behavioural factors between the different species, and clustering of species by sub-district of residence. For secondary case detection in RACD, cases were more likely to be febrile and resided within 100 m or in the same household as the index case.

The finding of a higher risk of infection in adult males is consistent with other studies reported from low-endemic areas [3, 15, 45–47]. The variation of age distribution between high- and low-endemic areas is presumably due to the acquisition of immunity after frequent exposure [4] as well as occupational and behavioural factors [46]. In Aceh, forest-based work such as: logging, rubber tapping, mining, and cannabis farming is usually performed by men. Moreover, these jobs require workers to stay overnight in the forest, which was found to be strongly association with malaria infection and affirming the finding from other studies that adult men are at high risk for infection in low-transmission settings due to occupation and behavioural factors.

A secondary aim was to identify risk factors associated with specific *Plasmodium* types. One strength of this study was the use of molecular testing to classify species. The programme at district level had only been using microscopy and 20 *P. knowlesi* cases were misdiagnosed as *P. falciparum*, *P. vivax*, *P. malariae*, and indeterminate. Because many of the *P. knowlesi* cases had not travelled, and due to the known presence of the pig-tailed macaque and *Anopheles leucosphyrus* [48], a known vector of *P. knowlesi*, in forested areas of Aceh Besar, local transmission was established. Detailed information about this discovery of *P. knowlesi* in this area has recently been described [21]. It is likely at that P*. knowlesi* transmission has been ongoing for some time, but missed by the programme due to the known challenge of *P. knowlesi* diagnosis by microscopy [49]. The local programme was keen to identify specific risk factors for infection by *P. knowlesi* in addition to the other *Plasmodium* types.

Consistent with the overall finding that infection was associated with work related overnights in the forest, all of the *P. falciparum* cases, and most of the *P. vivax* and *P. knowlesi* cases reported occupations that required overnight stays in the forest. However, *P. knowlesi* cases were more likely to have forest exposure due to residence or other activities and they were less likely to have travelled, suggesting infection acquisition related to non-work exposure as well. Infections were more likely in male adults for all species, though interestingly, the only three malaria cases in females and/or children were due to *P. knowlesi* and all had forest exposures. Others have reported women and children to typically be at lower risk of *P. knowlesi* given less forest exposure [50]. Certainly, one limitation of the risk factor assessment was that exposures to macaque monkeys was not assessed because *P. knowlesi* infections were not anticipated. Finally, the most notable species-specific finding was that the species clustered by sub-district, suggesting further entomological and epidemiological investigation specific to the subdistricts (e.g. a macaque population near Lembah Seulawah, where most of the *P. knowlesi* cases occurred) is warranted.

In low-endemic areas moving toward elimination, RACD is a standard and widely practiced activity. In the study area, RACD was initiated in 2010. One challenge of RACD is that the activities are resource intensive and often yield few cases [16]. Some studies have looked at factors associated with secondary-case detection and found that secondary cases were more likely to be male [10, 16], live in the same house or close to an index case [10, 15, 51], live in a receptive area [52], have travel history to an endemic area [10], be detected within 7 days of an index case being reported [15], be associated with an index case classified as a local case [15], be under 5 years of age [53], symptomatic [17], and have a history of malaria infection [16, 17]. Although the numbers of secondary cases from this study were limited, the findings that secondary cases were more likely male, have forest-related work that required overnights near or in forest, report recent travel, report fever in the previous 2 weeks, and reside in the same house or within <100 m from the home of the index, were similar to those from other studies. While all secondary cases were found within 100 m of the index case, they were not more likely to have other risk factors that would be associated with local transmission (e.g. lack of travel, close distance to a water body). They shared the same risk factors as index cases including recent travel, forest exposures, and occupations that required overnights in the forest, suggesting that infection was less likely due to local transmission in their village. Given these findings, the low yield of RACD (0.4 %, or 6/1495), the high level of resources required to conduct it, and the possibility that secondary cases might eventually present through passive surveillance anyway, it could be argued that efforts could be better spent directly targeting forest workers rather than just targeting villagers, and the screening radius for RACD in village could be decreased to 100 m, contrary to the WHO recommendation for a 1 to 2-km screening radius in RACD due to the potential flight range of *Anopheles* mosquitoes [13].

This study was performed to inform local malaria elimination planning. In order to target adult males with forest exposure, the District Health Office Aceh Besar could develop collaborations with other relevant government departments (e.g., forestry, agriculture, transmission, and forest squatter resettlement) and local partners (farm owners, logging site supervisors and farming companies) to engage forest workers and their supervisors in malaria elimination activities. These partners could support education campaigns to promote health-seeking behaviours. Also, although these interventions still need to be rigorously evaluated for *P. knowlesi*, they could also provide insecticide-treated hammocks or tarpaulins or ITNs [54, 55] which are often not available at the workplace because distribution by the government usually takes place at the residence and these products are not otherwise available for purchase in markets. Finally, with work requiring overnight stays in a forest location outside of the village or sub-district of residence being a major risk factor for infection, migration and mobility surveillance system linked to the existing malaria surveillance system could be developed for use across sub-district, districts and provinces.

The main limitation of this study was the small sample size, which limited the ability to rigorously analyse risk factors for species-specific infection and secondary case detection, and in the primary analysis, led to wide confidence intervals in the OR estimates. Small sample sizes are a common challenge for studies in low transmission or elimination settings. In the future, better anticipation of decline in cases could prompt inclusion of neighbouring districts to improve sample size, which would also facilitate understanding of human movement across districts and its impact on malaria transmission. Another potential limitation of this study was the cross-sectional design, which can only identify associations and not prove causality. Moreover, there may be unmeasured factors confounding the association of observed independent variables with the outcome variables.

Cohort studies are an ideal study design to provide evidence of relationship between exposure and outcome or disease before the disease occurred, yet these studies are expensive, time consuming, require large sample sizes, and have risk of loss to follow-up or withdrawal of participants. Case–control studies are useful for assessing rare condition, but there is the challenge of identifying the appropriate control population and potential for recall bias. Despite their limitations, cross-sectional surveys enable analysis of data that includes point or cumulative prevalence of disease, and they are often simple, inexpensive and rapid to perform [56]. Passive and active surveillance data are easily available in most settings, and uninfected subjects screened serve as a convenient control group for cross-sectional analyses.

The study had several additional strengths. Firstly, highly sensitive and specific molecular methods were utilized to confirm malaria infection and species identification. Second, this study is one of few that looks at risk factors for *P. knowlesi* [50, 57], which has only recently been discovered in Indonesia outside Borneo [21]. Additionally, it is the first undertaking in Indonesia, and among few studies from any setting, that explores the factors associated with secondary case detection in RACD [10, 15–17].

## Conclusion

Risk factor analyses in low-endemic areas aiming for malaria elimination are essential to inform targeting of interventions. The methodology and approach used in this study can be easily adapted to other settings with similar endemicity and passive and active surveillance programs in place. This study provided useful information on risk factors for malaria in Aceh Besar District, and will help to inform malaria elimination planning in Aceh Besar District and Indonesia at large. The high burden of *P. knowlesi* warrants further investigation into its epidemiology and specific risk factors in this setting, and highlights a new challenge for malaria elimination in endemic areas.

## Abbreviations

AIC: Akaike’s information criteria (AIC)
API: annual parasite incidence
DBS: dried blood spot
DNA: deoxyribonucleic acid
IRS: indoor residual spray
ITN: insecticide-treated net
LAMP: loop-mediated isothermal amplification
LLIN: long-lasting, insecticidal nets
LRT: likelihood ratio test
PCR: polymerase chain reaction
PHC: primary health centre
QA: quality assurance
RACD: reactive case detection
WBC: white blood cells
WHO: World Health Organization
